# Rainfall anomalies and typhoid fever in Blantyre, Malawi

**DOI:** 10.1017/S0950268822000759

**Published:** 2022-05-10

**Authors:** Jillian S. Gauld, Sithembile Bilima, Peter J. Diggle, Nicholas A. Feasey, Jonathan M. Read

**Affiliations:** 1 Institute for Disease Modeling, Bill and Melinda Gates Foundation, Seattle, Washington, USA; 2 Centre for Health Informatics, Computing, and Statistics, Lancaster University, Lancaster, UK; 3 Malawi-Liverpool Wellcome Research Programme, Kamuzu University of Health Sciences, Blantyre, Malawi; 4 Department of Clinical Sciences, Liverpool School of Tropical Medicine, Liverpool, UK

**Keywords:** Typhoid fever, rainfall, weather, statistical analysis

## Abstract

Typhoid fever is a major cause of illness and mortality in low- and middle-income settings. We investigated the association of typhoid fever and rainfall in Blantyre, Malawi, where multi-drug-resistant typhoid has been transmitting since 2011. Peak rainfall preceded the peak in typhoid fever by approximately 15 weeks [95% confidence interval (CI) 13.3, 17.7], indicating no direct biological link. A quasi-Poisson generalised linear modelling framework was used to explore the relationship between rainfall and typhoid incidence at biologically plausible lags of 1–4 weeks. We found a protective effect of rainfall anomalies on typhoid fever, at a two-week lag (*P =* 0.006), where a 10 mm lower-than-expected rainfall anomaly was associated with up to a 16% reduction in cases (95% CI 7.6, 26.5). Extreme flooding events may cleanse the environment of *S.* Typhi, while unusually low rainfall may reduce exposure from sewage overflow. These results add to evidence that rainfall anomalies may play a role in the transmission of enteric pathogens, and can help direct future water and sanitation intervention strategies for the control of typhoid fever.

## Introduction

Typhoid fever, caused by *Salmonella enterica* serovar Typhi, is a major cause of febrile illness in low- and middle-income countries, with 10–20 million cases occurring annually worldwide [1]. Available evidence indicates *S.* Typhi is a human restricted pathogen, however, it has been isolated outside of the human host, in both drinking water and sewage [2, 3]. Individuals can be exposed through close interaction with infected individuals via food handling or contamination of other fomites, known as short-cycle transmission. However, typhoid fever infection can also occur through exposure to the environment, such as through ingestion of contaminated drinking water or crops. This form of transmission is referred to as long-cycle transmission.

In many locations with ongoing transmission of *S*. Typhi, the specific mechanisms of long-cycle transmission are unknown, however in some locations, the mechanisms have been elucidated. In Chile, irrigation of crops with wastewater was identified as a risk factor for typhoid. After this practice was banned, typhoid incidence declined to near-elimination levels [4]. In Nepal, transmission through drinking water was posited, and further bolstered by environmental sampling [3]. Understanding these pathways is important for designing water and sanitation control measures that are likely to be necessary for the elimination of the disease alongside the typhoid conjugate vaccine [5, 6]. As climate is a key determinant of environmental conditions, the analysis of weather events, such as rainfall, on typhoid could help to identify factors which support both environmental survival and transmission in endemic locations. Further, if a link to a weather pattern is established, this may help to understand fluctuations in disease incidence.

Because both disease incidence and meteorology typically exhibit seasonal variation, evidence of a cross-correlation between the two does not establish a causal or mechanistic link. Typhoid is known to be seasonal [7], therefore it is unsurprising that unadjusted rainfall and disease incidence exhibit cross-correlation. In previously published analyses of this kind, in Dhaka, a 3–5 week lag in rainfall was associated with an increase in typhoid cases [8], whilst in a multi-site investigation, it was observed that rainfall often precedes the disease, and a positive association with temperature is frequent [7], but this was not a universal finding across the evaluated study sites.

Whilst time series analysis can be helpful in establishing causality, it is important to make a distinction between (a) association between rainfall and incidence and (b) association between rainfall and incidence *anomalies*, i.e. residuals about the expected values of both series at a given time of year. A statistical association between rainfall and incidence series, i.e. case (a) above, could arise simply because both exhibit seasonal variation, for whatever reason. An analysis of rainfall and incidence anomalies addresses the hypothesis that *unusual* rainfall patterns are associated with unusual levels of disease incidence. Although it is still the case that association does not automatically imply causality, a causal interpretation becomes more plausible, provided that the temporal lag of a statistical association is compatible with the incubation period of the disease in question, because it eliminates the possibility that the statistical association is simply a by-product of the mutual seasonality of the two series. This approach has previously been used in establishing a link between rainfall events and diarrhoea [9].

Since 1998, Queen Elizabeth Central Hospital in Blantyre, Malawi has conducted blood culture surveillance for typhoid fever. A sharp increase in reported cases occurred in 2011, the majority of which were multi-drug resistant [10]. Despite ongoing transmission, the mechanisms of transmission remain unknown. A risk factor study conducted in 2015 suggested complex interactions between environmental and common social exposures, including using river water for cooking and cleaning [11]. 59.6% of the population in Blantyre use non-flushing latrines [12], and it has been noted that the rocky soil in Blantyre often prevents the digging of pit latrines deeper than three metres [13], providing a hypothesis for a mechanistic link between heavy rainfall events and subsequent contamination of river water or the surrounding environment. In this setting, temperature and rainfall patterns were previously jointly explored in relation to typhoid fever, and a four-month lagged association with rainfall was found, along with the protective effects of excessive rainfall [14]. The goals of the current study expand upon this work to focus on rainfall anomalies: the association between rainfall and case incidence anomalies. We did this by using rainfall anomalies as an explanatory variable in a model for case-incidence and estimating the effects of rainfall anomalies, lagged within a biologically plausible range of 1–4 weeks, adjusting for both long-term trends and seasonal variation in case-incidence.

## Methods

### Hospital and rainfall data

Beginning in 1998, laboratory records from Queen Elizabeth Central Hospital for typhoid fever have been recorded. Cases of blood culture-confirmed *S.* Typhi, identified through routine hospital-based surveillance on both inpatients and outpatients, were recorded in a logbook (until 2010) or an electronic Laboratory Information Management System (from October 2010 onwards). We obtained weather data from the Malawi Meteorological Service, which included daily measurements of rainfall (mm) collected from Chichiri weather station in central Blantyre city. Due to reporting and laboratory time lags based on the day of the week, we summarised the data into weekly counts of cases and weekly average rainfall. All data processing and subsequent analyses were conducted in R version 3.5.1 [15], and a type 1 error rate was designated as 5%. From inspecting the data beginning in 1998, there was low transmission of typhoid prior to 2012, therefore analyses used data beginning 1 January 2012 to focus on the period of endemic transmission of the disease.

### Smoothing of typhoid case time series

We first modelled the time series of typhoid cases. Because we know typhoid cases are seasonal, and exhibited a large increase in 2011, we needed to incorporate both a seasonal term and a smooth time-trend. We did not attempt to explore any predictive drivers of the increase in 2011, as this has been explored previously through a dynamic modelling framework. That study attributed the rise in cases to an increase in shedding duration, possibly caused by multi-drug resistance [16]. We used a quasi-Poisson log-linear model, which allows us to model typhoid case-counts over time while accounting for over-dispersion. We use the penalised regression spline (the default in *mgcv* package for the R statistical programming language) and an annual seasonal harmonic.

### Modelling rainfall and defining anomalies

In order to capture rainfall patterns and define anomalies, we needed to be able to predict an ‘expected’ amount of rainfall throughout our study period. We utilised a joint model with two components. First, we modelled the amount of rain on days with rainfall using a log-linear model, log(*rain*(*t*)) = *m*(*t*) + ϵ(*t*), where ϵ(*t*) is a residual series, Normally distributed with mean zero and variance *σ*^2^, and *m*(*t*) includes annual and six-month harmonic terms to describe the seasonal effect [equation 1].1
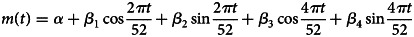


The six-month harmonic terms in [1] were needed to capture the asymmetric shape of the seasonal variation. Next, we modelled the probability of rainfall, *p*(*t*), in any given week using logistic regression, including the same annual and six-month harmonic terms [equation 2].2
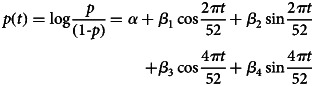


The expectation of total rainfall on any given day is then the product of the probability of any rain and the conditional expectation of the amount of rain on a day with rain, hence:3
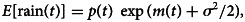
where *σ*^2^ is estimated from the fitted rainfall model [equation 1] and the exponential term follows from the properties of the log-Normal distribution. A rainfall anomaly was then defined, for each week in the study period, as the observed rainfall minus the expected rainfall.

### Exploring seasonal relationships

We examined cross-correlations of average weekly rainfall and typhoid fever cases, in order to characterise seasonal trends in relation to weather events in the raw data. Cross-correlations were generated between de-trended case counts, retaining the seasonal component, and average weekly rainfall, for lags spanning 0–24 weeks.

We then aimed to estimate the lag between the seasonal peaks of case incidence and rainfall. We generated 1000 realisations of model parameters using the multivariate Normal sampling distribution of the parameter estimates for the fitted typhoid case and rainfall models. We then extracted the timing of the seasonal peaks for cases and rainfall from model predictions using these parameters. Finally, we took the difference in seasonal peaks for each set of realisations to estimate the lag between cases and rainfall. We summarised the lag in terms of mean and 95% confidence interval (CI).

### Relating typhoid and rainfall anomalies

To explore the relationship between rainfall and typhoid anomalies, we used a quasi-Poisson log-linear model [equation 4].
4
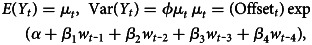
where *w*_*s*_ is the rainfall anomaly for week *s*.

This model accounted for the overall trend in cases by using the fitted typhoid case model, which includes both seasonal and time-trend components, as an offset term. By using these model predictions as an offset in the descriptive model, we are able to estimate the extent to which lagged rainfall anomalies act as a dampener or booster of the current transmission level, with a view to obtaining evidence in support of a causal hypothesis, as discussed earlier.

We included terms for rainfall anomalies, as defined above, at lagged weeks 1 to 4. This range of lags was informed by the known incubation period of typhoid (typically 7–14 days) [17], and accommodates for potential delay in healthcare-seeking and case identification. We explored potential relationships between rainfall anomalies and case anomalies using the model [equation 4], in which the rainfall anomaly effects are log-linear, as well as explored a log-quadratic relationship.

We evaluated the overall contribution of the 1 to 4 week lagged rainfall anomalies to the incidence model using a Wald test [18], which provided an indication of whether the included model parameter estimates were significantly different from zero.

## Results

### Typhoid case time series model

**Fig. 1.**
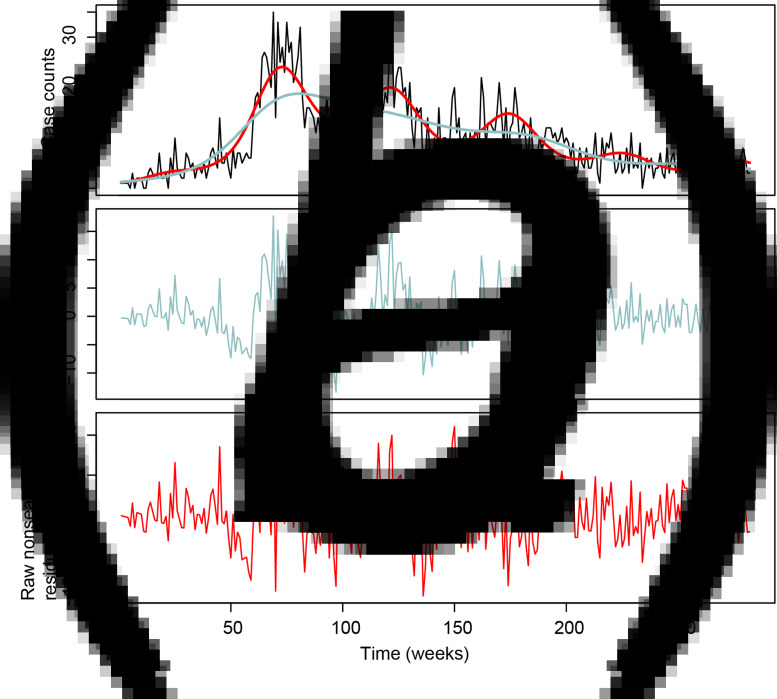
(a) time series of case-counts (black), with long term trend (blue) and long term plus seasonal trend (red). (b) Residuals from long-term trend model. (c) Residuals from long term plus seasonal trend model.

**Fig. 2.**
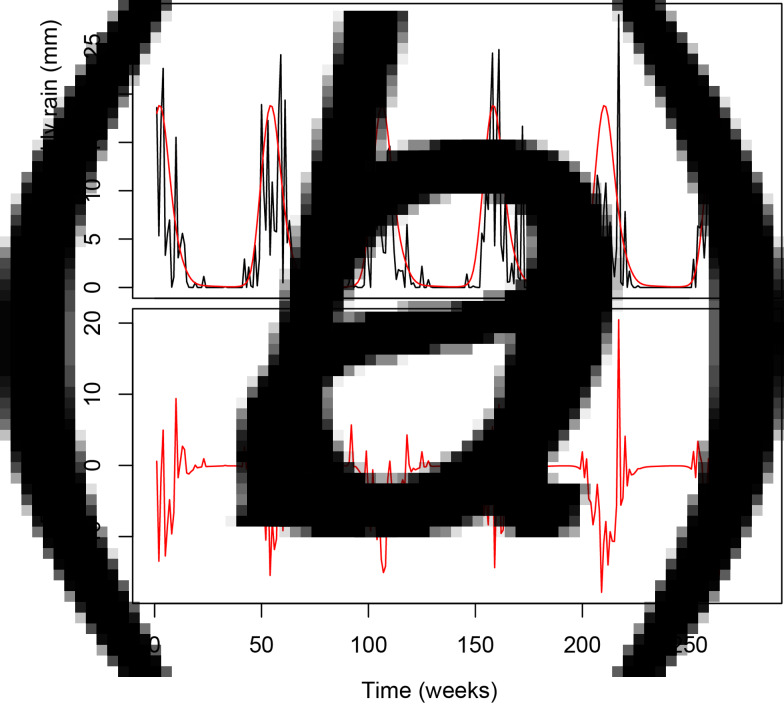
(a) Average weekly rainfall (black), with fitted log-Gaussian model (red). (b) Rainfall anomalies.

The typhoid case model with and without seasonal components is shown in Figure 1a. The de-trended seasonal case-counts are shown Figure 1b, and the de-trended, de-seasonalized residuals are shown in Figure 1c, representing typhoid anomalies with and without the seasonal component, respectively. The fit to our joint model for the occurrence and amount of expected weekly rain is shown in Figure 2a. We used this model to generate the rainfall anomaly sequence as observed minus expected weekly rain (Fig. 2b).

### Seasonal relationships between cases and rainfall

**Fig. 3.**
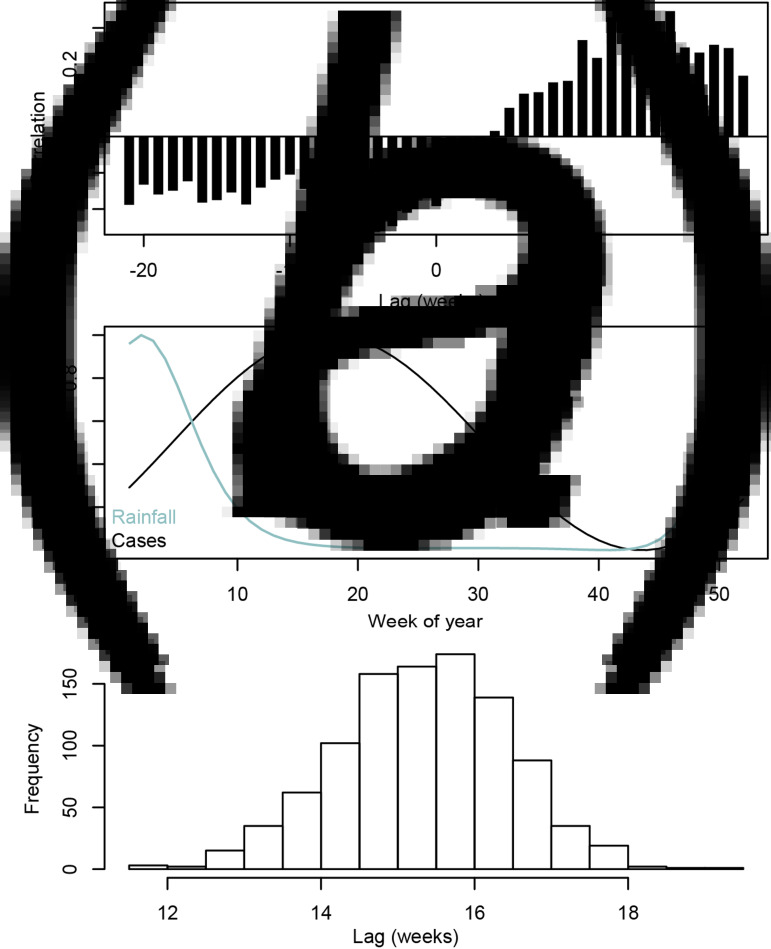
(a) Cross-correlation of detrended cases and rainfall, (b) Best-fit seasonal amplitude for cases (black line) and rainfall (blue line), (c) Histogram of the calculated seasonal lags generated from 1000 realisations of the multivariate Normal distribution parameterised by model covariates.

Correlations between detrended case counts, retaining the seasonal trend (Fig. 1b), and rainfall were calculated, and are shown in Figure 3a. Rainfall is positively correlated with case counts at lags between approximately 10 and 20 weeks (Fig. 3a). Additionally, there is a lag between the seasonal pattern of fitted rainfall and case model predictions over a single year (Fig. 3b). The estimated lag between the peak rainfall and cases was 15.46 weeks (95% CI 13.28, 17.65).

### Relationship between case and rainfall anomalies

**Fig. 4.**
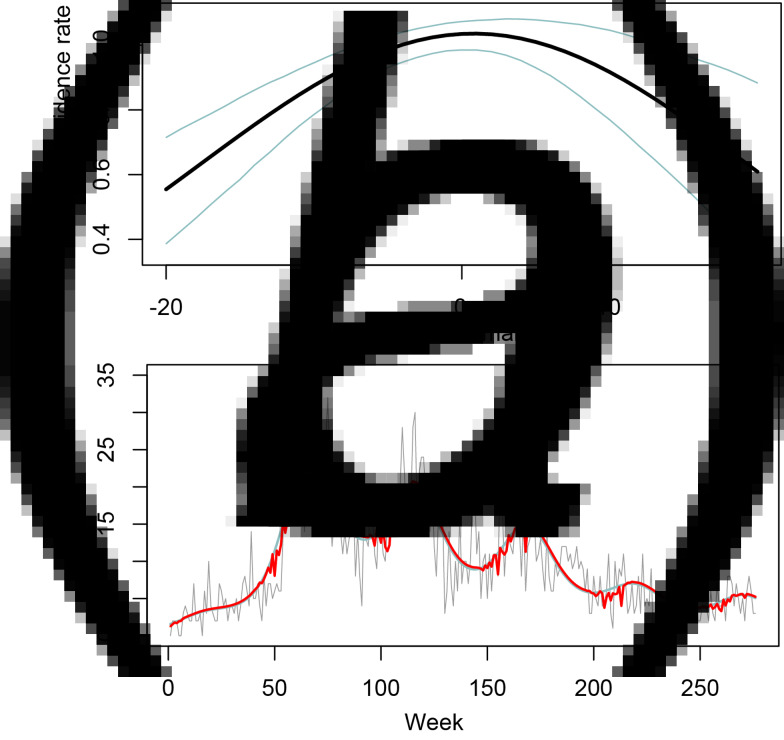
(a) Effect of 2-week lagged rainfall anomaly on case incidence, (b) Model predictions with (red) and without (blue) rainfall anomaly included, and total cases in light grey.

We did not find a significant overall association between rainfall anomalies and incidence anomalies when assuming a log-linear relationship [Wald test, *P* = 0.18]. Results from the model assuming a log-quadratic relationship were flagged for further evaluation [Wald test, *P* = 0.05]. Investigating the log-quadratic model further, we identified the association was significant at the 0.01 level at a lag of two weeks [*P* = 0.006, Table 1]. We refitted the model including only the 2 week-lagged linear and quadratic coefficient, resulting in a significantly improved fit of the model to the data compared with the null model (likelihood ratio test: scaled deviance = 11.46, degrees of freedom = 2, *P* = 0.003, Table 2). The model suggests rainfall anomalies with extremely lower or higher rainfall than expected are associated with reduced typhoid case incidence (Fig. 4).

**Table 1.** Summary of estimates from log-quadratic model with all lags included

Coefficient	Value	Standard error	*P*
Intercept^a^	0.023	0.027	0.402
*w*_*t*−1_	0.004	0.006	0.445
*w*_*t*−2_	0.008	0.006	0.170
*w*_*t*−3_	0.004	0.005	0.497
*w*_*t*−4_	−0.002	0.005	0.727
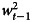	0.0002	0.0005	0.622
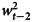	−0.002	0.0006	0.006
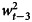	−0.0003	0.0005	0.472
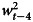	0.001	0.0005	0.144

a *w*_*s*_ represents the rainfall anomaly for week *s*.

**Table 2.** Summary of the quadratic rainfall anomaly model including only the two-week lag

Coefficient	Value	Standard error	*P*
Intercept^a^	0.039	0.025	0.123
*w*_*t*−2_	0.007	0.005	0.165
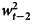	−0.001	0.0005	0.005

a *w*_*s*_ represents the rainfall anomaly for week *s*.

## Discussion

The pathway between shedding of *S*. Typhi and subsequent ingestion by another exposed individual is poorly understood. The primary reservoir of *S*. Typhi is humans, however it must survive in the environment sufficiently to permit transmission to the next human host. Rainfall may act as a mediator in this process. A large increase in typhoid fever cases occurred in Blantyre, Malawi in 2011, and was associated with an increase in multi-drug resistance [10, 16]. The current study focuses not on these long-term trends, but fluctuations in cases due to environmental interactions such as rainfall. Specifically, in this study, we aimed to explore the relationship between rainfall anomalies and typhoid anomalies in Blantyre, Malawi.

Daily rainfall and typhoid case incidence both exhibit seasonal patterns in Blantyre. We found the peak in rainfall precedes the peak in cases by approximately 15 weeks, consistent with previous work in this setting [14]. As the incubation period of typhoid fever is typically between 1 and 4 weeks [17, 19], this is not supportive of rainfall being a primary driver of typhoid incidence in Blantyre without an unknown intermediate step or steps. It is unclear what bio-social mechanism could generate the long lag between total rainfall and typhoid incidence we observe in Blantyre. However, when two processes are seasonal, they inevitably will exhibit significant correlation at one or more time-lags. When incorporating weather events as predictive processes, constraining lagged effects by known biological processes is critical for interpretation.

We therefore explored the possibility of deviation from the seasonal pattern of rainfall – rainfall anomalies – to describe typhoid cases at biologically plausible lags of 1 to 4 weeks. We found a significant non-linear association between rainfall anomalies and typhoid case anomalies, with the highest typhoid incidence associated with the seasonally expected rainfall. In translating the coefficients to their impact on case counts, we found that a 10 mm lower-than-expected rainfall anomaly was associated with up to a 16% reduction in cases (95% CI 7.6, 26.5).

If rainfall is a mechanism that disseminates *S*. Typhi by facilitating exposure of susceptible humans, for example through flooding of pit latrines or runoff of sewage into rivers used for drinking water or domestic use [11], it is plausible that a drier than expected period could reduce typhoid case incidence. Conversely, more rainfall than expected may have a cleansing effect on *S*. Typhi in the environment, through dilution or removal of the pathogen from river water sources. After an anomalous rainfall event, individuals may be at a lower risk of developing disease through dilution of the inoculum of *S*. Typhi to a level below that which would be expected to cause typhoid fever, a level which has been quantified through Typhoid live challenge models [17, 20].

The protective effect of heavy rainfall has been reported for other enteric diseases, including diarrhoeal disease in Ecuador [9]. Further, recent global burden estimates for typhoid found the proportion of the population living in the monsoon belt was a significant predictor of incidence, indicating that these extreme events may put individuals at higher risk of typhoid fever [1]. However, these data are based on large-scale global models, and do not include rainfall as a time-resolved variable.

Rainfall anomalies are distinct from the overall seasonal pattern of rainfall, which we found was not correlated with typhoid cases within biologically plausible lags and therefore was not included in our descriptive model. This poses a question for further investigation, as it appears that both extremely lower or higher rainfall than expected is associated with reduced typhoid incidence. Better understanding the biological and environmental responses to seasonally expected *vs.* anomalous rainfall would aid in further interpretation of these results.

There are some limitations to this study. The time series of typhoid cases reflects the date of blood culture diagnosis of a patient. However, the time at which an individual is infected precedes this by the incubation period, and to a lesser extent by individual variations in treatment seeking. Whilst a range of incubation periods have been reported for typhoid in human challenge studies [17], they infrequently exceed the two-week lagged effect found in our study. This is therefore a biologically plausible lag that is keeping with the pathogenesis of typhoid. The geographic span of Blantyre (approximately 20 km) may indicate differential propensities to seek care based on the distance to the hospital. Our time series does not, therefore, precisely represent the date of infection. Further, we do not have a precise estimate of *S*. Typhi transmission due to short-cycle transmission (transmission independent of the environment) at the time of this study, and this may impact the signal from rainfall and environmental interactions. However, from previous studies of typhoid risk in Blantyre, environmental factors were identified as major risk factors and therefore likely play a significant role in transmission in this setting [11].

Overall, this study identified a complex relationship between rainfall and typhoid fever incidence. We found an extended lag between the seasonal patterns of typhoid fever and rainfall in Blantyre, Malawi, though this is likely spurious association lacking a biological mechanistic explanation. Additionally, we found evidence that rainfall anomalies are associated with reduced typhoid case incidence. Improved data could help strengthen these observations, including prioritising the detection of typhoid cases closer to their time of exposure through active surveillance, and improved environmental sampling and detection to understand the distribution of *S*. Typhi in the environment and over time, and human exposure and infection risk. Further work to explore these relationships in other locations, and better understand the ecological niches of *S*. Typhi, will help advance our understanding of the link between weather patterns and typhoid transmission.

## Data Availability

Data used for this study is available in the Supplementary Materials.

## Supplementary material

For supplementary material accompanying this paper visit http://doi.org/10.1017/S0950268822000759. click here to view supplementary material
